# Description of an extant salamander from the Gulf Coastal Plain of North America: The Reticulated Siren, *Siren reticulata*

**DOI:** 10.1371/journal.pone.0207460

**Published:** 2018-12-05

**Authors:** Sean P. Graham, Richard Kline, David A. Steen, Crystal Kelehear

**Affiliations:** 1 Department of Biology, Geology, and Physical Sciences, Sul Ross State University, Alpine, Texas, United States of America; 2 School of Earth, Environmental, and Marine Sciences, University of Texas Rio Grande Valley, Brownsville, Texas, United States of America; 3 Georgia Sea Turtle Center, Jekyll Island Authority, Jekyll Island, Georgia, United States of America; 4 Smithsonian Tropical Research Institute, Apartado, Ancon, Panama, Republic of Panama; Universita degli Studi della Tuscia, ITALY

## Abstract

The salamander family Sirenidae is represented by four extant species that are restricted to North America. Sirens are abundant throughout the southern United States and are among the world’s largest amphibians, yet the biology, ecology, and phylogeography of this group is poorly-known. In this study we use morphological and genetic evidence to describe a previously unrecognized species from southern Alabama and the Florida panhandle. We name this species the Reticulated Siren, *Siren reticulata*. Future studies will enable more precise phylogenetic information about *S*. *reticulata* and will almost surely reveal additional undescribed species within the family.

## Introduction

Extant members of Sirenidae are completely aquatic, eel-like salamanders with an unusual morphology: large fimbriate external gills and only front limbs. Their ancestors likely branched off from all other salamanders early in the evolution of this group [1]. Although Sirenids ranged through North America, South America, and Africa during the Cretaceous [2], the family is now largely restricted to the southern United States and northeastern Mexico [3], where it has been present since the late Eocene [4].

The family Sirenidae (Gray 1825) contains two long-recognized extant genera: *Pseudobranchus* (Gray 1825) and *Siren* (Linnaeus 1766), separable by the number of toes on the forelimb (three in *Pseudobranchus*, four in *Siren*) and number of gill slits (one in *Pseudobranchus*, three in *Siren* [3]); herein we focus on the latter genus, with two currently-recognized species. The Lesser Siren (*S*. *intermedia* Barnes 1826) reaches 30–69 cm in length and reportedly occurs throughout much of the southern United States, into Mexico, and along the Mississippi River watershed to Michigan [3]. At nearly 1 m in length, the Greater Siren (*S*. *lacertina* Linneaus 1766) is one of the largest salamanders in the world; its geographic range is generally confined to portions of Atlantic and Gulf Coastal Plains of the southeastern United States from Maryland to Alabama, including all of Florida [3].

The two recognized *Siren* species can be difficult to distinguish largely because there is considerable overlap and variation in morphology, ecology, and geographic range [3, 4]. Partially due to a general lack of information about siren natural history and the fact that they can be found within a number of isolated river drainages, authors have noted for decades that multiple additional species from this group are likely to be formally described pending ongoing genetics analyses [3]. However, to date little has been published.

In this study, we formally describe a previously unrecognized sirenid species. As initially noted by Mount [5], this animal’s morphology is sufficient to distinguish it from other currently recognized sirens; our formal genetic and morphometric analyses strongly support its recognition. This species is genetically distinct from all currently-recognized species and subspecies of *Siren* and co-occurs with at least one of them. This species is very large (maximum known size nearly 60 cm), making it among the largest vertebrates described from the United States in over 100 years. In the process of describing this species, we hope to bring attention to how poorly understood this group of fascinating animals is as well as inspire researchers and funding institutions to prioritize rigorous work on the basic biology, ecology, and phylogenetics of Sirenidae.

## Materials and methods

Based on museum records, this species was first collected 15 April 1970 from the Fish River in Baldwin County, Alabama (AUM 18547); Mount [5] specifically mentioned this specimen by noting it did “not conform” to descriptions of *S*. *lacertina*. Over nine years after the Fish River specimen was collected, two comparable specimens were collected incidentally while trapping turtles from Lake Jackson near Florala, Covington County, Alabama (R. Mount, T. Lamb, and D. Vogt; AUM 27972–73). The purported species was also observed but not collected years later in Conecuh National Forest, Covington County, Alabama [6,7].

**Fig 1:**
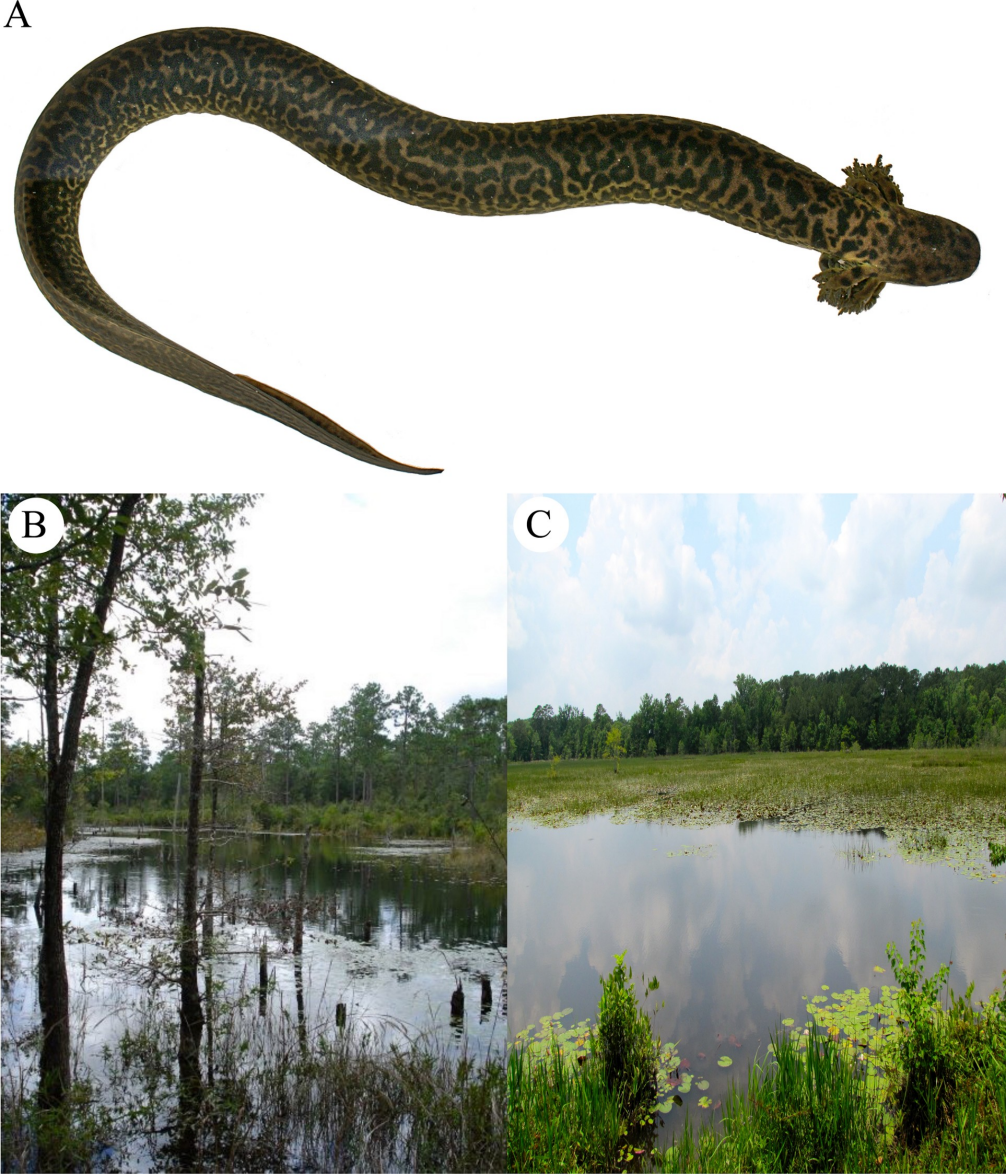
(A) *Siren reticulata* paratype specimen captured in Okaloosa County, Florida. (B) Location of *Siren reticulata* captured in 2009 by D. Steen and M. Baragona. (C) The type locality of *Siren reticulata*, Walton County, Florida.

On 4 September 2009, D. Steen captured a single individual of the purported *Siren* species while trapping turtles within Eglin Air Force Base, Okaloosa County, Florida ([8]; AUM 39360; Fig 1). At this point the authors began dedicated efforts to collect more specimens. Subsequent sampling on Eglin Air Force Base failed to procure additional specimens and efforts to procure specimens in Conecuh National Forest and in Lake Jackson also failed. However, on 8 June 2014, three more specimens were collected in a freshwater marsh adjacent to Lake Jackson in Walton County, Florida. Morphological analyses and/or genetic material from the specimens noted above as well as *S*. *intermedia* and *S*. *lacertina* from several other populations enabled diagnosis of the undescribed species. Herein we use the names of currently recognized species and acknowledge that future analyses are likely to split these taxonomic entities.

### Morphological analysis

We primarily followed Hanlin and Mount [9] for morphological measurements. Using vernier calipers (accurate to the nearest 0.1 mm) we measured: snout-vent length (SVL), tail length, head length (maximum straight-line distance from the anterior-most snout to the first branch of the gills), trans-orbital width (interorbital width, including the eyes), head width (width of the head at its widest point), and mid-trunk width (width of trunk across midline at a point halfway down the body). We also counted the number of costal grooves from the axial region to the posterior vent on the right and left side of the body. We compared tail length, head length, trans-orbital width, head width, mid-trunk width, and number of costal grooves among *S*. *intermedia intermedia*, *S*. *i*. *nettingi*, *S*. *lacertina*, and the new species using ANCOVA with SVL as the covariate. To determine where the differences lay we used Tukey’s honest significant difference (HSD) posthoc analyses; significant differences are denoted by uppercase letters in the results. We compared head measurements among siren specimens (head width, head length, and trans-orbital width) using a principal components analysis. All statistical analyses were performed using JMP pro version 9.0.0. Genetic data were not available for museum specimens of *S*. *intermedia* and *S*. *lacertina* used for morphological comparisons.

### DNA extraction, PCR, and analysis

All specimens were collected and handled in accordance with Institutional Animal Care and Use Committee (IACUC) protocols at the University of Texas Rio Grande Valley (2013-005-IACUC Kline) and Auburn University (2013–2386). *Siren* DNA for the purported undescribed species, *S*. *intermedia* (Arkansas), and *S*. *intermedia* (east Texas) was extracted from ethanol preserved tissues using a DNA extraction kit (GenCatch Blood & Tissue Genomic Mini-Prep Kit; #1460050, Epoch Life Science Sugar Land, TX, USA). For nuclear genome analysis, extracted DNA was diluted and sent to Harvard Medical School Biopolymers Lab for library preparation and Illumina sequencing. For mitochondrial sequence analysis, three overlapping mitochondrial fragments of expected sizes: 5693 Kb, 5733 Kb and 6887 Kb were amplified by polymerase chain reactions (PCR) using 50 ml reaction volumes consisting of GoTaq Long PCR Master Mix (Promega Corp., Madison WI), 50 ng DNA with *Siren*-specific primers and PCR conditions detailed in LaFortune ([10]; Table 1). PCR products were run in 0.7% agarose TBE electrophoresis, stained with ethidium bromide, and visualized with a blue LED transilluminator. Bands were excised with a clean scalpel, then purified with a gel extraction kit (GenCatch Gel Extraction Kit #2260050, Epoch Life Science Sugar Land, TX, USA). Fragments were quantified using fluorescence (Quant-iT dsDNA Assay Kit, high sensitivity # Q33120, Thermo Fisher Scientific, USA) and then combined at equivalent dilutions. Combined fragments for each sample were sent to Harvard Medical School Biopolymers Lab for library preparation and Illumina Next Seq.

**Table 1:** List of mtDNA primers used to amplify sequence mitochondrial DNA from the three amplicons from LaFortune [10].

Fragment Name	Primer Name	Sequence 5’ → 3’
L1-C2	**L1-FW-1**	ACACCGCCCATCACCCTCA
	**C2-RV-2 **	CCCTGCTAACCCTAAGAAATGTTGTGG
C2-G	**C2-FW-1 **	TGTCTTGTCTATGGGGGCAGTATTTGC
	**G-RV-2INT **	GGCGTGTCATCAGCCAATTA
G-L1	**K9FW-GTOL1 **	ACCCTCTTTACAAACCGAGAAGG
	**K10RV-GTOL1**	CATCCCACTCTTTTGCCACAG

Sequenced libraries were processed in Geneious V11 (Gene Codes, Ann Arbor, MI). Reads were paired using the Paired Read function in Geneious, Illumina adapters were removed using BBDuk and reads below 30 bp were discarded. Paired reads were merged using BBMerge at the lowest sensitivity setting. Merged reads >100 bp were assembled to reference sequences from *S*. *intermedia texana* (KU904486) and *S*. *lacertina* (KU904488) for mitochondrial gene sequences for 12S, and 16S cytochrome oxidase 1 (COI), NADH dehydrogenase 5 (ND5) and cytochrome b CYB. A minimum coverage cutoff of 30 was used in assemblies and consensus sequences were based on the majority nucleotide at each position.

For the nuclear genome, de novo assemblies were conducted using 19,634,162 reads from the *S*. *intermedia* AR library in CLC Genomics Workbench. Default settings were used with reads mapped back to contigs (mismatch cost = 2, insertion cost = 3, deletion cost = 3, length fraction = 0.5, similarity fraction = 0.8). The join contigs function of the Genome Finishing Module plug-in was then used to combine overlapping contigs. The sequences obtained from the assemblies included the external transcribed spacer intron (ETS), 18S, internal transcribed spacer 1 intron (ITS-1), 5.8S, internal transcribed spacer 2 intron (ITS-2), and 28S with greater than 33-fold coverage. The published sequence for *Xenopus laevis* (X02995) was aligned and used for annotation. The annotated *S*. *intermedia* AR sequence was then used as a reference sequence for assembly of sequences for all other sirens used in analysis.

Because the largest database of published genetic sequences for *Siren* are from Cytochrome B, these sequences were used as a first step to clarify the position of Sirens used in this study compared to all published CYB sequences, especially those from the only two formally identified species: *S*. *lacertina* and *S*. *intermedia*. A Blast search in Genbank was performed to find all available *Siren* mitochondrial CYB sequences and a CYB sequence from *Pseudobranchus axanthus* was included as an outgroup (Table 2). Alignment of the sixteen sequences was conducted in Muscle 3.8.425 [11] then all aligned CYB sequences were trimmed to the shortest fragment size of 770 nucleotides. PartitionFinder 2.1.1 [12] was used to determine the best partitioning scheme for the sixteen *Siren* CYB sequences + outgroup according to the Akaike information criterion (AICc). A maximum likelihood phylogeny was conducted in RAxML 8.2.11 using each codon position as a separate partition and GTR+G substitution model for 1,000 bootstraps.

**Table 2:** Samples and GenBank accession numbers used for genetic analysis in this study; species names conform to currently recognized taxonomy although some specimen identities are likely to change pending comprehensive phylogenetic analyses of Sirenidae.

Sample	Locality	Description	18S-28S	12S	GenBank 16S	Accession ND5	Numbers COI	CytB	Publication
*Pseudobranchus axanthus*	Florida	Everglades dwarf siren	-	GQ368660	GQ368660	GQ368660	GQ368660	GQ368660	[1]
*Siren intermedia* (AR)	Arkansas	*Siren intermedia* isolate 55RK	MH806872	MH888029	MH888029	MH888033	MH888032	MH888034	This study
*Siren intermedia*	Louisiana	*Siren intermedia*	-	GQ368661	GQ368661	GQ368661	GQ368661	GQ368661	[1]
*Siren intermedia* 169	Florida	*Siren intermedia* haplotype 169	-	-	-	-	-	AY713288	[17]
*Siren intermedia* 168	Florida	*Siren intermedia* haplotype 168	-	-	-	-	-	AY713287	[17]
*Siren intermedia* 167	Florida	*Siren intermedia* haplotype 167	-	-	-	-	-	AY713286	[17]
*Siren intermedia* (TX)	Texas	*Siren intermedia*	-	-	-	-		MH806871	[18]
*Siren intermedia* (E. TX)	Texas	*Siren intermedia* isolate 53RK	MH888026	MH888024	MH888024	MH888024	MH888024	MH888024	This study
*Siren intermedia* (TX)	Texas	*Siren intermedia* isolate S49RK	MH888027	-	-	-	-	-	This study
*Siren intermedia* (TX)	Texas	*Siren intermedia* isolate S56RK	MH888028	-	-	-	-	-	This study
*Siren intermedia texana* 1	Texas	*Siren intermedia texana* haplotype 1	-	KU904486	KU904486	KU904486	KU904486	KU904486	[10]
*Siren intermedia texana* 2	Texas	*Siren intermedia texana* haplotype 2	-	-	-	-	-	KU904484	[10]
*Siren intermedia texana*		*Siren intermedia texana* isolate S52RK	MH888025	-	-	-	-	-	This study
*Siren lacertina*	Florida	Greater siren	MH806871	KU904488	KU904488	KU904488	KU904488	KU904488	[10]
*Siren lacertina* 173	Florida	*Siren lacertina* haplotype 173	-	-	-	-	-	AY713292	[17]
*Siren lacertina* 171	Florida	*Siren lacertina* haplotype 171	-	-	-	-	-	AY713290	[17]
*Siren lacertina* 170	Florida	*Siren lacertina* haplotype 170	-	-	-	-	-	AY713289	[17]
*Siren lacertina* 172	Florida	*Siren lacertina* haplotype 172	-	-	-	-	-	AY713291	[17]
*Siren* sp.	Florida	*Siren* sp.	-	-	-	-	-	AY691720	[18]
*Siren reticulata*	Florida	Reticulated siren isolate 50RK	MH806870	MH888035	MH806874	MH806873	MH888031	MH888030	This study
*Xenopus laevis*	-	African clawed frog	X02995	-	-	-	-	-	-

A second analysis was conducted using *S*. *intermedia nettingi*, *S*. *i*. *intermedia*, *S*. *lacertina*, the purported undescribed species, and *P*. *axanthus* as the outgroup (Table 2). Alignments of gene sequences for 12S, 16S, COI, ND5, and CYB were conducted in Muscle 3.8.425 [11]. The sequence data was partitioned by gene and further partitioned by codon position for COI, ND5, and CYB. PartitionFinder 2.1.1 [12] was used to determine the best partitioning scheme and nucleotide substitution models for each gene according to the Akaike information criterion (AICc). MrBayes 3.2.6 [13] was used to estimate phylogenetic relationships, under the partitioning models determined by PartitionFinder (Table 3). Bayesian analysis was run for five million generations, sampled every 1000 generations, with 25% of the generations discarded as burn-in. Four Markov chain were used with default heating parameters. The analysis was repeated twice to assess the robustness of the posterior probabilities. The concatenated sequence alignments were also used to build a maximum likelihood tree in RaxML. PartitionFinder 2.1.1 [12] was used to determine the best partitioning scheme and nucleotide substitution model according to the Akaike information criterion (AICc). A maximum likelihood phylogeny was conducted in RAxML 8.2.11 using rapid bootstrapping (i.e.1000 bootstraps) and search for best-scoring ML tree using the–f a–x 1 option.

**Table 3:** Best nucleotide substitution models for *Siren* and *Pseudobranchus axanthus* outgroup using five gene sequences: 12S, 16S, COI, ND5, and CYB determined by PartitionFinder V2.1.1. The models of evolution include gamma distributed rate variation among sites (G) and the proportion of invariable sites (I).

Subset	Partition Names	Best Model
1	ND5_1stpos	GTR+I
2	ND5_2ndpos	GTR+I
3	ND5_3rdpos	GTR+G
4	12S,CYTB_1stpos	GTR+G
5	CYTB_2ndpos	GTR
6	CYTB_3rdpos	HKY+G
7	COI_1stpos	GTR+I
8	COI_2ndpos	F81+I
9	COI_3rdpos	GTR+I
10	16S	GTR+I+G

An analysis of the 18S to 28S nuclear sequence was conducted using six siren taxa with *Xenopus laevis* (X02995) as an outgroup. The 18S, 5.8S, and 28S sequences showed little variability between any *Siren* samples and were excluded from further analysis. Therefore, the externally transcribed spacer (ETS), internally transcribed spacer I (ITS-1), and internally transcribed spacer II (ITS-2) were aligned separately in Muscle 3.8.425 [11]. All alignments were concatenated and individual sequence units were considered as separate data partitions. The best partitioning scheme of three separate sequences was determined by PartitionFinder 2.1.1 [12] using the Akaike information criterion (AICc; Table 4). MrBayes 3.2.6 [13] was used to estimate phylogenetic relationships. Bayesian analysis was run as described for the mtDNA analysis. The concatenated sequence alignments were also used to build a maximum likelihood tree in RaxML RAxML 8.2.11 using each sequence as a separate partition and GTR+G substitution model for 1,000 bootstraps.

**Table 4:** Best nucleotide substitution models for *Siren* and *Xenopus laevis* outgroup using three nuclear sequences: externally transcribed spacer (ETS), internally transcribed spacer I (ITS-1) and internally transcribed spacer II (ITS-2) determined by PartitionFinder V2.1.1. The models of evolution include gamma distributed rate variation among sites (G).

Subset	Partition Names	Best Model
1	ETS	GTR
2	ITS-1	GTR+G
3	ITS-2	HKY+G

### Nomenclatural acts

The electronic edition of this article conforms to the requirements of the amended International Code of Zoological Nomenclature, and hence the new name contained herein is available under that Code from the electronic edition of this article. This published work and the nomenclatural acts it contains have been registered in ZooBank, the online registration system for the ICZN. The ZooBank LSIDs (Life Science Identifiers) can be resolved and the associated information viewed through any standard web browser by appending the LSID to the prefix “http://zoobank.org/”. The LSID for this publication is: urn:lsid:zoobank.org:pub ED852CEE-52EE-40B6-9A9D-1F03B801E4B2. The electronic edition of this work was published in a journal with an ISSN and has been archived and is available from the following digital repositories: PubMed Central, LOCKSS.

## Results

### Holotype

In life, the holotype was a 39.7 cm SVL, 61.1 cm total length, 221 g female collected by SPG, DAS and CK on 08 June 2014 and deposited in the Auburn University Museum of Natural History (AUM 40669). Head length = 35.7 mm; trans-orbital width = 17.1 mm; head width = 24.2 mm; mid-trunk width = 34.3 mm; costal grooves = 40. This specimen contains hundreds of tiny (< 1 mm) developing follicles. Although slightly darker overall in coloration compared to the smaller, paratype specimen depicted in Fig 1A, the holotype adheres closely to the description that follows.

### Paratypes

Paratypes are five formalin-preserved specimens in the Auburn University Museum of Natural History (AUM 18547, 27972–73, 39360, 40668) and one specimen housed in the Museum of Vertebrate Zoology, University of California, Berkeley (MVZ 269554). At least one of these is an adult male of similar size to the holotype (AUM 40668; 40.6 cm SVL). Mean morphological measurements of the paratypes and holotype (N = 7) are presented in Table 5.

**Table 5:** Morphological data for sirens collected in Alabama, Georgia, Louisiana, Arkansas, and Florida. Means ± 1 S.E. (minimum-maximum).

	*Siren intermedia intermedia*	*Siren intermedia nettingi*	*Siren lacertina*	*Siren reticulata*
Sample size*	38	57	18	7
Snout-vent length (cm)	14.8 ± 0.6 (9.8–24.0)	23.2 ± 0.6 (12.1–31.9)	35.8 ± 1.9 (18.1–53.0)	33.4 ± 1.9 (26.5–41.5)
Mid trunk width (mm)	12.3 ± 0.6 (7.7–21.7)	20.8 ± 0.6 (9.2–29.0)	36.7 ± 2.1 (13.9–47.7)	31.8 ± 2.5 (24.0–38.4)
Tail length (cm)*	7.5 ± 0.4 (1.0–12.5)	11.8 ± 0.3 (6.1–18.4)	17.1 ± 1.0 (9.4–24.5)	19.1 ± 1.1 (15.6–22.2)
# costal grooves	33.8 ± 0.4 (29.0–39.5)	34.9 ± 0.2 (31.5–37.5)	38.3 ± 0.5 (30.5–41.0)	40.5 ± 0.3 (39.0–41.5)
Head width (mm)	12.5 ± 0.6 (6.4–22.6)	20.9 ± 0.6 (10.8–27.6)	35.1 ± 2.4 (16.4–56.3)	24.2 ± 1.3 (19.4–29.1)
Head length (mm)	19.7 ± 0.9 (12.4–33.2)	29.9 ± 0.7 (16.0–39.5)	50.4 ± 2.3 (29.0–67.4)	36.1 ± 1.6 (29.6–43.7)
Trans-orbital width (mm)	8.7 ± 0.4 (5.7–18.3)	13.7 ± 0.4 (7.2–19.8)	20.5 ± 1.1 (8.2–26.7)	16.4 ± 1.1 (12.6–20.5)

* Ten specimens had broken tails; therefore *n* = 34 for *S*. *intermedia*; *n* = 54 for *S*. *lacertina nettingi*; *n* = 17 for *S*. *lacertina*; *n* = 5 for *S*. *reticulata* for tail length.

### Type locality

A shallow (< 1m depth) marsh near Florala, Alabama, on the Walton County, Florida portion of Lake Jackson (Fig 1C). The marsh is separated from the open water of the lake to the northeast by swamp and a low dike and road. The marsh is heavily vegetated; aquatic floating and emergent plants such as white water lily (*Nymphaea*), water shield (*Brasenia schreberi*), pickerel weed (*Pontaderia*), and cattail (*Typha*) cover much of the wetland.

### Etymology

This animal has been colloquially referred to as the Leopard Eel. However, given that the species is neither a leopard nor an eel, we selected Reticulated Siren as a more appropriate formal common name. The specific name, *reticulata*, is a reference to the reticulated pattern typical of all specimens we examined.

Siren reticulata, sp. nov.

Reticulated Siren

urn:lsid:zoobank.org:act:1411CA74-BEB3-4C72-9CA2-9DE4304582AD

### Diagnosis

Like all Sirenids, *S*. *reticulata* has an elongate, eel-like body shape, two forelimbs, no eyelids, a lateral line, enlarged external gill fimbriae associated with gill slits, and a horny beak in place of the premaxillary teeth typical of other salamanders. There are only two known genera in the family Sirenidae: *Pseudobranchus* and *Siren*. The genus *Pseudobranchus* (dwarf sirens) includes two species (restricted to Florida, southern Georgia, and South Carolina) and is diagnosed by the presence of only one gill slit and three digits on each limb. The species we describe herein is assigned to the genus *Siren* based upon its large size, presence of four digits on the forelimbs, and three permanent gill fimbriae with three associated external gill slits. The dorsum of *S*. *reticulata* is olive-grey with lighter yellow-green flanks. It has an obvious and striking dark reticulate spotted pattern beginning at the gill arches and continuing to the tail (Fig 1A). Some specimens show a decided boundary where the spotting pattern ends along the flanks, while others show continuous spotting along the flanks that continue onto the ventral surface. The venter is a lighter olive green-yellowish color and in some specimens, it is also sparsely covered with irregular spots.

Several morphological features distinguish *S*. *reticulata* from *S*. *intermedia* and *S*. *lacertina*. First and most obviously is the color pattern. *S*. *intermedia* is usually dark or light grey with no evidence of spotting, and if spots are present, they are usually small, sparsely distributed, and round. Specimens of *S*. *lacertina* are often nondescript greenish, dark grey, or black, but they can have green or gold flecks that are typically small in size and sparsely distributed on the dorsum. The patterning of both *S*. *intermedia* and *S*. *lacertina* is often lost during preservation. By contrast, *S*. *reticulata* has numerous, large, dark irregular spots in a reticulate patchwork that covers the dorsal surface completely. This pattern is evident in preserved specimens collected over forty years ago.

Specimens of *S*. *intermedia* are typically smaller than the *S*. *reticulata* specimens we collected, with costal grooves numbering 31–38 [3]. *Siren reticulata* is apparently one of the largest species of extant amphibians (mean = 33.4 cm SVL), but it is unknown whether it can achieve such sizes as *S*. *lacertina*. Costal grooves number 38–42 (mean 40.5) in *S*. *reticulata*, versus 30–41 (mean 38.3) in Alabama *S*. *lacertina* ([5]; Table 5). The new species has the highest costal groove count of any siren species (and presumably, the highest number of trunk vertebrae; ANCOVA SVL: F_1,115_ = 21.67; p <0.0001; species: F_3,115_ = 10.58; p < 0.0001; Tukey’s HSD indicates *S*. *reticulata* has more costal grooves than any other species; *S*. *lacertina*, *S*. *i*. *intermedia*, and *S*. *i*. *nettingi* do not differ from one another).

Our analysis of various morphological measurements revealed additional significant differences between siren species (see Table 5 for mean values and ranges; significant differences between species are indicated below by different uppercase letters after each species name). Snout vent length differed with species: *S*. *reticulata* and *S*. *lacertina* did not differ in SVL but both were significantly larger than *S*. *i*. *nettingi* and *S*. *i*. *intermedia*, which were in turn significantly different from one another (F_3,116_ = 85.90; p <0.0001). Mean head length varied significantly among species (SVL: F_1,115_ = 261.05; p < 0.0001; species F_3,115_ = 20.69; p < 0.0001; Tukey’s HSD *S*. *lacertina* A, *S*.*i*. *intermedia* B,C, *S*. *i*. *nettingi* B, *S*. *reticulata* C), indicating that *S*. *lacertina* has the largest head relative to its body size, whereas *S*. *reticulata* has a relatively small head. Significant differences in head width among species indicated that *S*. *reticulata* has a relatively narrow head (SVL: F_1,115_ = 281.02; p < 0.0001; species F_3,115_ = 14.68; p <0.0001; Tukey’s HSD *S*. *lacertina* A, *S*. *i*. *intermedia* A, *S*. *i*. *nettingi* A, *S*. *reticulata* B) and a narrow trans orbital width (SVL: F_1,115_ = 227.72; p < 0.0001; species: F_3,115_ = 5.20; p = 0.002; Tukey’s HSD *S*. *lacertina* A, *S*. *i*. *nettingi* A, *S*. *i*. *intermedia* A,B, *S*. *reticulata* B). Further, the first principal component of head width, head length, and trans orbital width explained 96.3% of the variance in head shape parameters and varied significantly among species (mean of first principal component = SVL: F_1,115_ = 367.54; p < 0.0001; species: F_3,115_ = 16.05; p < 0.0001; Tukey’s HSD = S. *lacertina* A, *S*. *i*. *nettingi* B, *S*. *i*. *intermedia* B, *S*. *reticulata* C).

These results underscore the general impression we observed in specimens of *S*. *reticulata*: in gross appearance it has a smaller, narrower head for its size compared to *S*. *intermedia* or *S*. *lacertina*. We also found differences between species in mid trunk width, with *S*. *lacertina* having the broadest trunk (SVL: F_1,113_ = 225.67; p < 0.0001; species F_3,113_ = 5.84; p = 0.001; Tukey’s HSD = *S*. *lacertina* A, *S*. *reticulata* A,B, *S*. *i*. *nettingi* B, *S*. *i*. *intermedia* B), and tail length, with *S*. *reticulata* having the longest tail (excluding all 10 specimens with a broken tail, SVL: F_1,105_ = 192.36; p < 0.0001; species: F_3,105_ = 5.35; p = 0.001; Tukey’s HSD = *S*. *reticulata* A, *S*. *i*. *nettingi* B, *S*. *i*. *intermedia* B, *S*. *lacertina* B). Additional morphological comparisons with larger sample sizes that can incorporate sex, geographic location, and age as covariates will likely provide additional pertinent information.

### Genetic analysis

**Fig 2:**
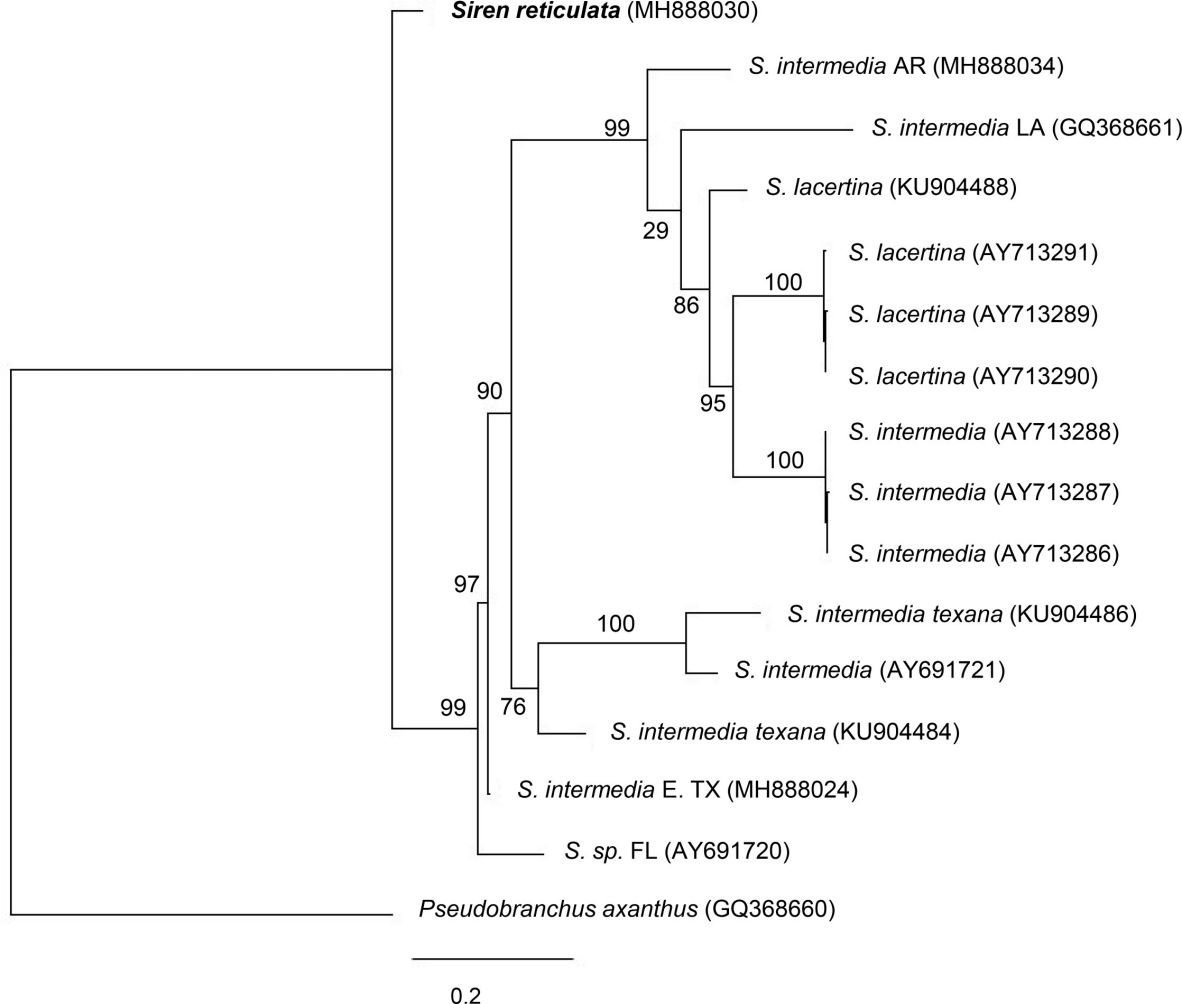
Maximum likelihood tree of Cytochrome B fragment (CYB) produced in RaxML using sequences from this study, all available published siren sequences, and *Pseudobranchus axanthus* as an outgroup. Node values are % of 1,000 bootstraps. For additional information, refer to Table 2.

The maximum likelihood analysis for all available CYB 770 nucleotide sequence fragments resulted in a tree showing that the two recognized species *S*. *lacertina* and *S*. *intermedia* fall in the same clade with 76% bootstrap support for *S*. *reticulata* sister to all *Siren* species for which published sequences are available (Fig 2). The closest taxa in the tree by genetic distance were a juvenile specimen incorrectly identified as *P*. *axanthus* (sample # AY691720; Table 2; Fig 2) collected from Gainesville Florida (Carl Franklin UT Arlington Museum personal communication) and a *Siren* from Jasper county, Texas (this study; see Table 2; Fig 2). The sequences for all identified *S*. *lacertina* were genetically distant from *S*. *reticulata* and formed a clade that contained all *S*. *lacertina* from Florida and *S*. *intermedia* from Florida, Arkansas, and Louisiana, indicating Florida *S*. *lacertina* may be more closely related to eastern populations of *S*. *intermedia* than to other *S*. *intermedia* subspecies to the west. Additionally, all *S*. *intermedia* from southern Texas formed a separate clade that was similarly distant from *S*. *reticulata*.

**Fig 3:**
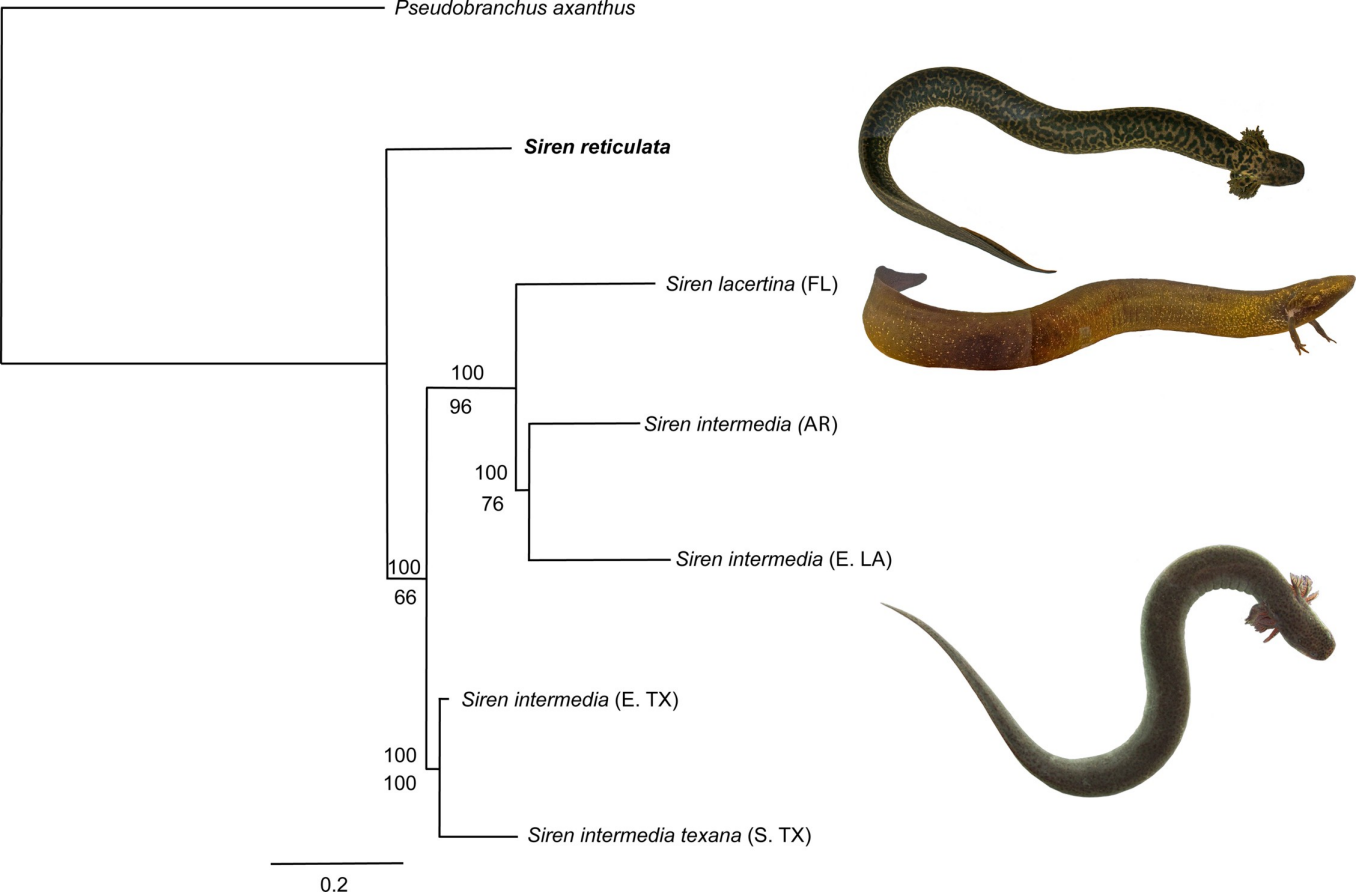
Bayesian inference consensus tree for sirens and *Pseudobranchus axanthus* outgroup using five gene sequences: CytB, COI, ND5, 12S and 16S. The tree was generated in MrBayes and run for 5 million generations using a partition scheme determined using Partition Finder 2. The top number is probability generated in MrBayes. The bottom number is the maximum likelihood % from 1,000 bootstraps generated in RAxML. Accession numbers are in Table 2.

The phylogenies of mtDNA gene sequences for 12S, 16S, COI, ND5, CYB using BA and ML analyses were concordant (Fig 3). The phylogenies showed that *S*. *reticulata* is not a direct sister taxon to any described species but is distant from them (*S*. *lacertina* and *S*. *intermedia*; Fig 3). This was confirmed in two independent runs resulting in 100% probability at each node. The maximum likelihood tree produced with RAxML yielded a concordant tree organization with moderate bootstrap support (66%) for *S*. *reticulata* sister to all other sirens in the dataset (Fig 3). As seen in the CYB phylogenies, the *S*. *lacertina*/*S*. *intermedia* group from Arkansas, eastern Louisiana and Florida differed markedly from the clade for sirens from east Texas and south Texas.

**Fig 4:**
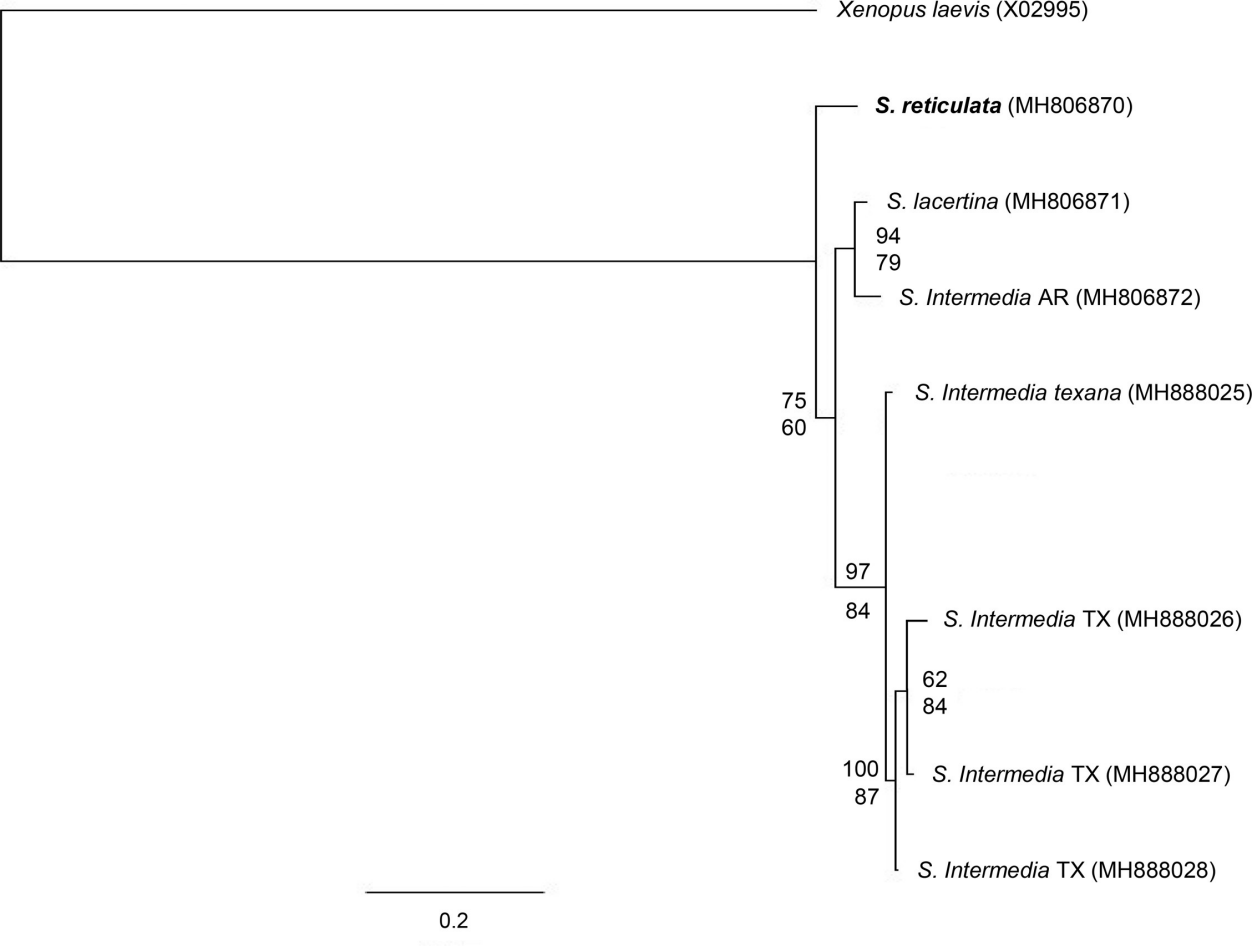
Bayesian inference consensus tree for sirens and *Xenopus laevis* outgroup using three nuclear gene sequences: IGS, ITS-1, and ITS-2. The tree was generated in MrBayes and run for 5 million generations using a partition scheme determined using Partition Finder 2. The top number is probability generated in MrBayes. The bottom number is the maximum likelihood % from 1,000 bootstraps generated in RAxML. For additional information, refer to Table 2.

The analysis of nuclear gene sequences for ETS, ITS-1, and ITS-2 in MrBayes resulted in a tree concordant with the mitochondrial phylogenies and with a 75% probability for *S*. *reticulata* as sister to all other known Siren species (Fig 4). The maximum likelihood tree was concordant but had low bootstrap support of 60% (Fig 4). Similar to the mitochondrial analyses, the *S*. *lacertina*/*S*. *intermedia* group were distant from the clade for sirens from east Texas and south Texas in the nuclear analysis as well.

### Distribution and habitat

To date this species is only confirmed from three localities. The type locality is a shallow freshwater marsh associated with a large freshwater limesink lake (Lake Jackson) that straddles the border between Florida and Alabama, USA, near the town of Florala, Alabama. AUM collection records indicate that *S*. *reticulata* has also been collected from the main body of Lake Jackson on the Alabama side of the lake. The second site is a beaver-impounded clearwater stream and associated bay swamp on Eglin Air Force Base, Okaloosa County, Florida (Fig 1B). Finally, the Fish River locality is a blackwater stream and associated bottomland forest in Baldwin County, Alabama [5].

**Fig 5:**
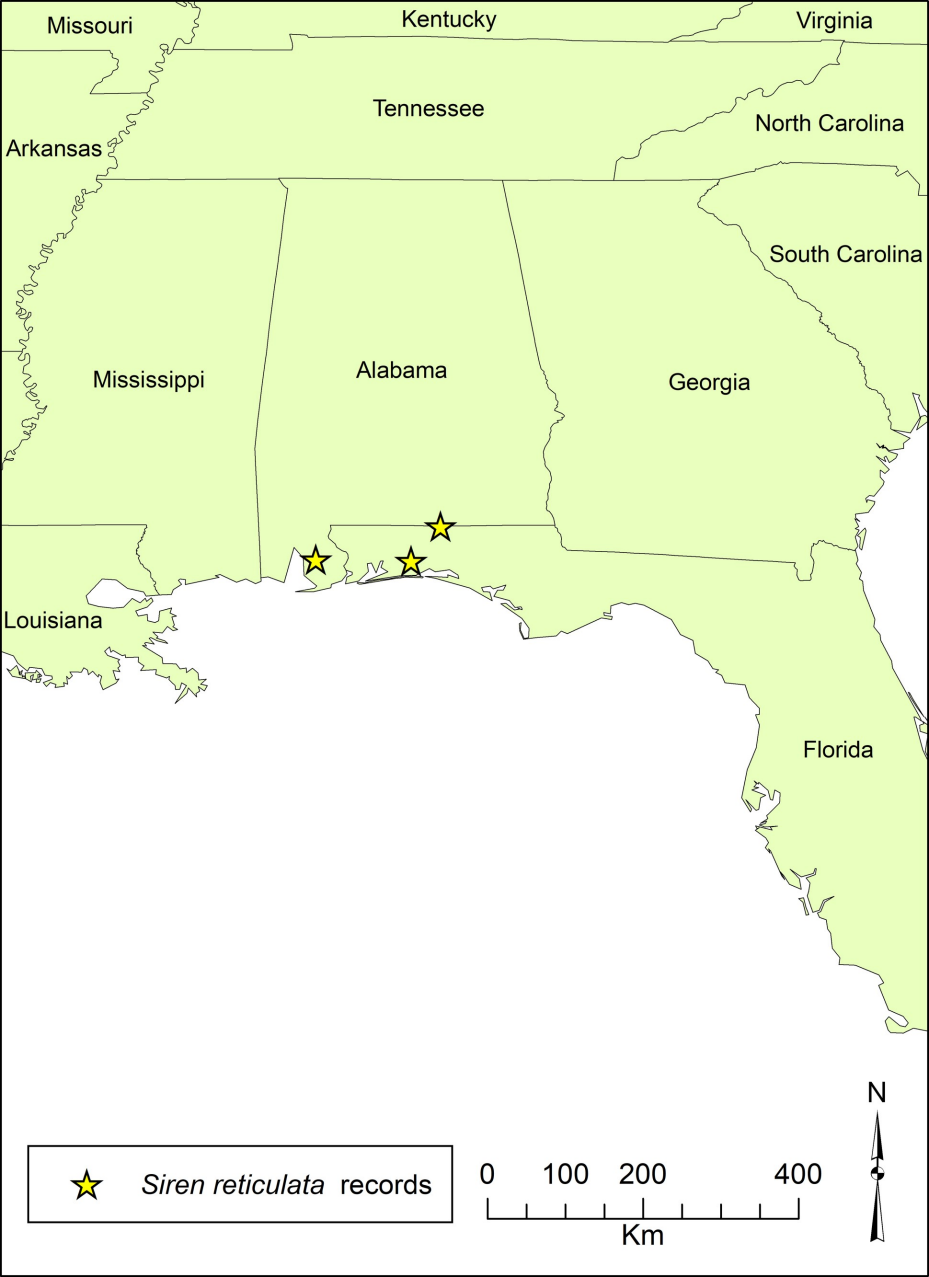
Known localities for *Siren reticulata* in the southeastern United States.

These very scant locality data suggest that *S*. *reticulata* occupies a diversity of freshwater habitats from the Mobile Bay-Tensaw Delta region of Baldwin County, Alabama to Okaloosa County, Florida and Covington County, Alabama (Fig 5). Records for *S*. *lacertina* immediately to the east of Florala in both Geneva and Henry County, Alabama indicate that *S*. *reticulata* and *S*. *lacertina* are at least parapatric in this region. We speculate that *S*. *reticulata* is endemic to the Panhandle region of Florida and Alabama and potentially southwestern Georgia, a globally-significant region because of its high species richness and endemism [14].

### Life history and ecology

Due to the difficulty in acquiring specimens and this species’ apparently limited distribution, little is known of *S*. *reticulata* life history and ecology. Clearly, research on this topic is an urgent need. Most of what we know of this species is consistent with the general habits of other described species of sirens. The holotype contains hundreds of tiny developing follicles, suggesting that females have high fecundity, a feature also exhibited by *S*. *lacertina*. Mating, fertilization mode (e.g., external or internal), nests, and eggs are undescribed. However, we emphasize that this information is scarce even for long-recognized *S*. *lacertina* or *S*. *intermedia*.

## Discussion

We present genetic and morphological evidence supporting the recognition of *Siren reticulata*, a heretofore-undescribed salamander in the family Sirenidae from Alabama and the Panhandle region of Florida. *Siren reticulata* is the first species of its family described since 1944 and it is among the largest species described from the United States over the last 100 years.

In this study we use morphological and molecular data (mitochondrial and nuclear sequences) to show that *S*. *reticulata* is a separate species from *S*. *lacertina* and all other taxa currently recognized as *S*. *intermedia*. We found strong support in the Bayesian analysis to place *S*. *reticulata* at the base of all known taxa in the genus *Siren*. However, the maximum likelihood model provided a lower level of support. Additional sampling and sequencing is required to refine the placement of *S*. *reticulata* and all other members in the genus *Siren* within the *Siren* phylogeny. For example, it is possible, given the alternating similarities in coding regions, that *S*. *reticulata* could be placed at the base of the “Eastern” clade of *S*. *lacertina*/*S*. *intermedia* or at the base of the *S*. *intermedia/S*. *nettingi* clade. This study also revealed that there are possibly several other undescribed *Siren* taxa, based on their position in the phylogenies. As has been surmised for decades, more work is needed to understand the diversity of the genus *Siren*. Beyond recognition of *S*. *reticulata* as unique, our results strengthen earlier suggestions that diversity of the genus is imperfectly understood and we invite more studies using a variety of molecular approaches (e.g., allozymes, mitochondrial and nuclear DNA sequences, and genomic approaches).

Sirens represent a large, important, and potentially diverse component of the vertebrate biomass in North American freshwater ecosystems [15] yet there have been no extensive phylogeographic analyses for this genus and we know little of their ecology and conservation. We chose to publish this description now, despite not having a complete understanding of the phylogenetic placement of *S*. *reticulata*, for several reasons. First, it has been decades since the first individual was collected and suspected to be an undescribed species; clearly this long delay has set back our understanding of these animals. Second, the species apparently occurs within a global biodiversity hotspot [14]. Given that much of their known habitats include wetlands embedded within the imperiled longleaf pine ecosystem [16] it is possible that *S*. *reticulata* is of conservation concern and it is difficult to afford formal protections to species that are not formally recognized. Finally, efforts to survey these animals, analyze genetic samples, and describe species have been sporadic due to the dearth of formally-funded studies. We hope the data we present here inspire others to prioritize further study of this group of fascinating amphibians and fund associated research.

## Supporting information

S1 Table Specimens examined as a component of the current study. Species names conform to currently recognized taxonomy although some specimen identities are likely to change pending formal and comprehensive phylogenetic analyses of Sirenidae.
(XLSX)

S1 Fig A siren from northwestern Florida (Walton County) captured and photographed by Pierson Hill. This specimen, although not included in our analyses, shows the typical Siren reticulata colour pattern.
(JPG)

S2 Fig A siren from northwestern Florida (Okaloosa County) captured and photographed by Pierson Hill. This specimen, although not included in our analyses, shows the typical Siren reticulata colour pattern.
(JPG)
