# Supplementary figures and images for: Description of an extant salamander from the Gulf Coastal Plain of North America: The Reticulated Siren, Siren reticulata
Source: PLoS One. 2018 Dec 5;13(12):e0207460. doi: 10.1371/journal.pone.0207460 (PMC6281224; doi:10.1371/journal.pone.0207460)

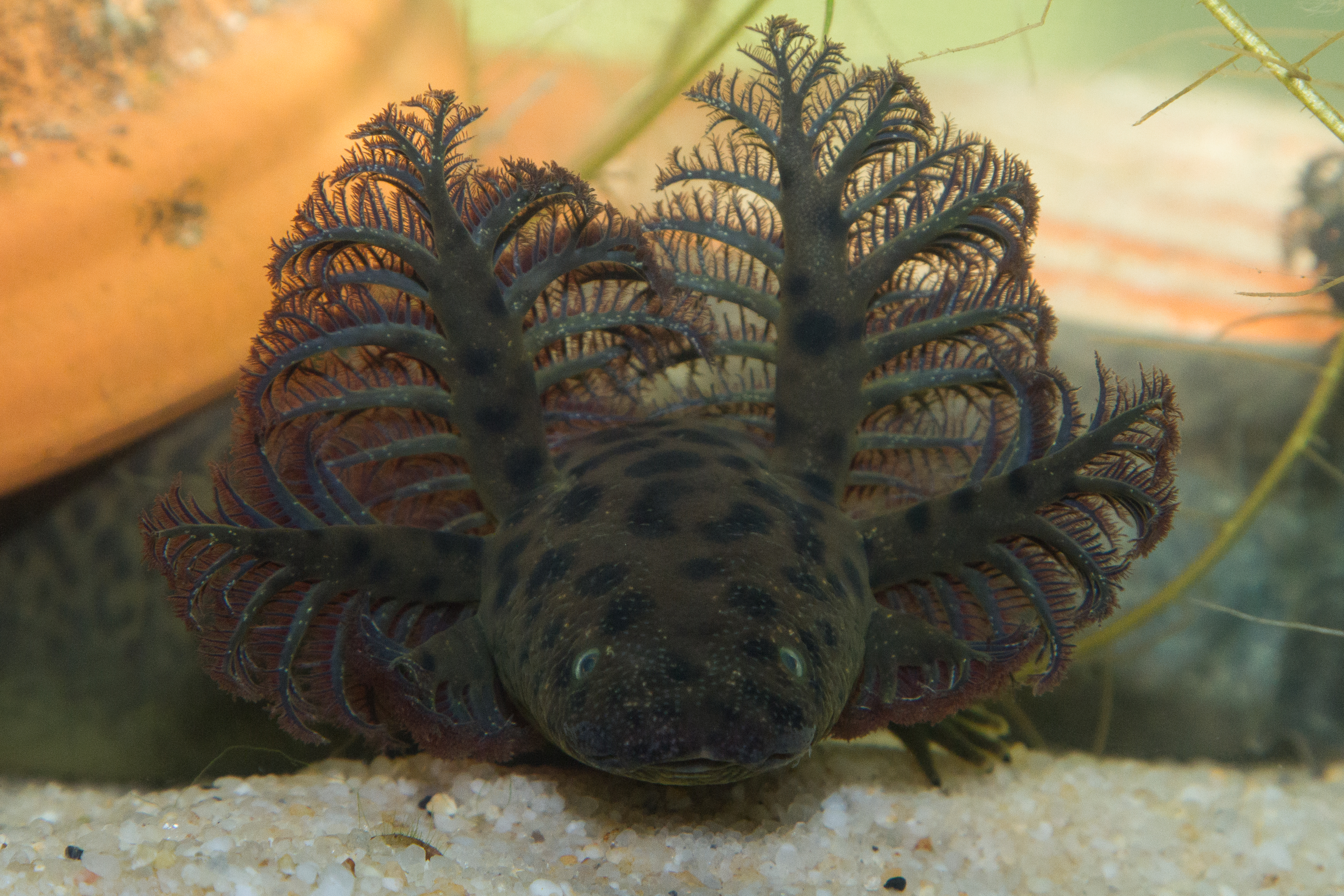

Supplement: S1 Fig — This specimen, although not included in our analyses, shows the typical Siren reticulata colour pattern. (JPG) [file pone.0207460.s002.jpg]

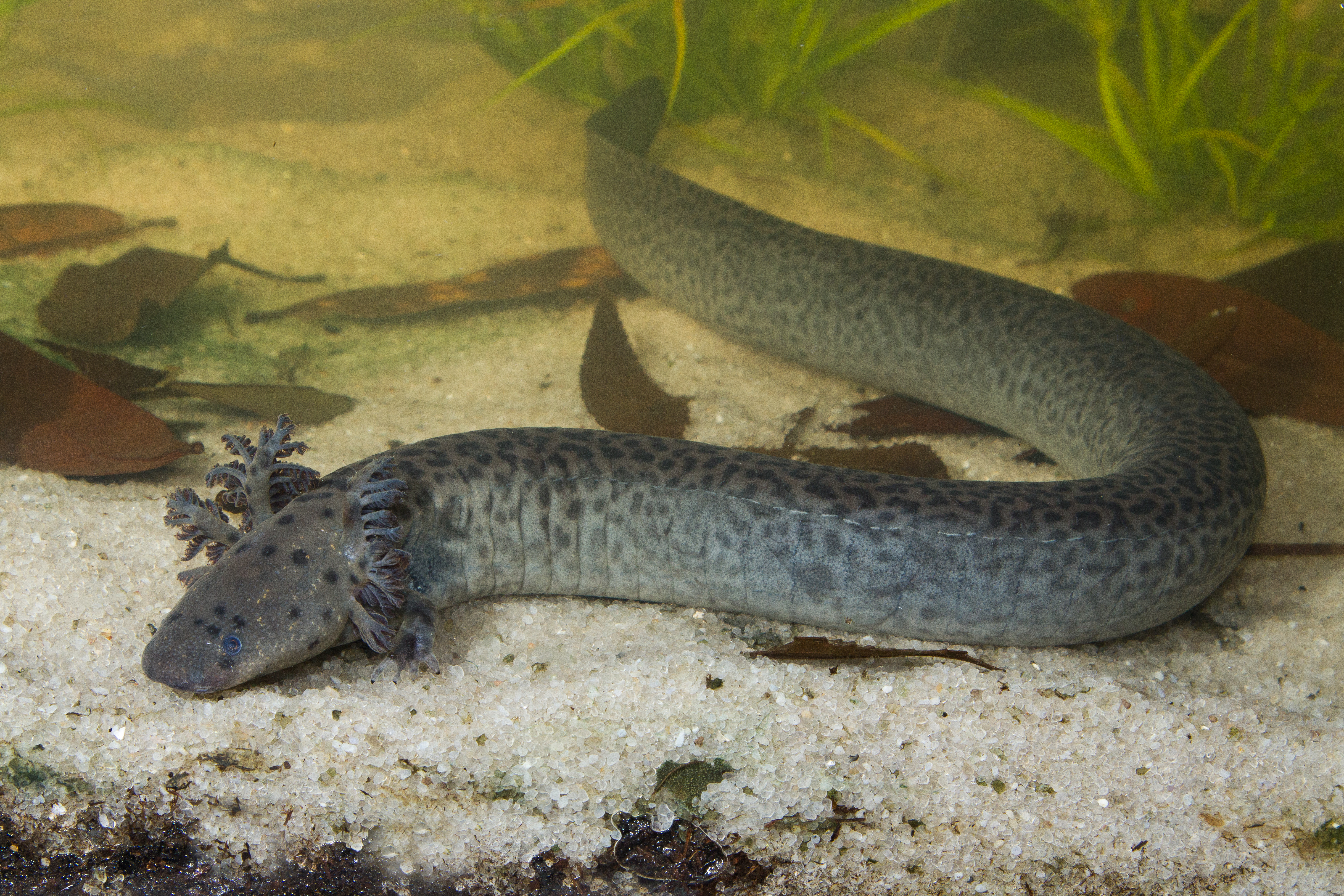

Supplement: S2 Fig — This specimen, although not included in our analyses, shows the typical Siren reticulata colour pattern. (JPG) [file pone.0207460.s003.jpg]
